# Are the energy matrix values of the different feed additives in broiler chicken diets could be summed?

**DOI:** 10.1186/s12917-020-02600-3

**Published:** 2020-10-15

**Authors:** Abdallah E. Metwally, Ahmed A. A. Abdel-Wareth, Ahmed A. Saleh, Shimaa A. Amer

**Affiliations:** 1 Department of Nutrition and Clinical Nutrition, Faculty of Veterinary Medicine, Zagazig University, Zagazig, 44511 Egypt; 2 Department of Animal and Poultry Production, Faculty of Agriculture, South Valley University, Qena, 83523 Egypt; 3 Department of Poultry Production, Faculty of Agriculture, Kafr Elsheikh University, Kafr Elsheikh, 33516 Egypt

**Keywords:** Broiler chickens, Growth performance, Energy matrix value, Natural feed additives

## Abstract

**Background:**

The aim of this study is to investigate whether the energy matrix values of the nonstarch polysaccharide- (NSP-) degrading enzymes, bioemulsifier (LYSOFORTE^®^), guanidinoacetic acid (CreAMINO^®^), or their combinations could be summed. The effects of these additives on the growth performance, carcass traits, and economic value of the broiler chicken diets were evaluated. A total of 525-one-day-old Ross chicks with an initial body weight of 42.96 ± 0.87 g were haphazardly allocated into seven groups with five replicates. The seven experimental treatments are as follows: (1) basal diet with no additives (breeder recommendation), which is the control group, (2) basal diet minus 100 kcal/kg supplemented with 0.02% NSP-degrading enzymes (NSP), (3) basal diet minus 50 kcal/kg supplemented with 0.025% emulsifier (LYSOFORTE^®^), (4) basal diet minus 50 kcal/kg supplemented with 0.06% guanidinoacetic acid (CreAMINO^®^), (5) basal diet minus 150 kcal/kg supplemented with a mixture of NSP and LYSOFORTE^®^ (NSPL), (6) basal diet minus 100 kcal/kg supplemented with a mixture of NSP and CreAMINO^®^ (NSPC), and (7) basal diet minus 200 kcal/kg supplemented with a mixture of NSP, LYSO, and CreAMINO^®^ (NSPLC). The experiment lasted for 35 days.

**Results:**

It was found that the final body weight, body weight gain, and relative growth rate were significantly higher in birds fed diets supplemented with NSPL, NSPC, CreAMINO, and LYSO with the reduced energy matrix value. The overall feed conversion ratio was significantly improved due to the supplementation of NSPC, CreAMINO, NSPL, and LYSO with the reduced energy matrix value compared to the control group. Moreover, no significant effect on the carcass criteria was observed by the different treatments. As a result of the dietary supplementation with NSPL, NSPC, CreAMINO^®^, and LYSO with the reduced energy matrix value, the net profit, total return, economic efficiency, and performance index were increased and the cost of feed per kg of body weight gain was decreased.

**Conclusion:**

The energy matrix value of NSPL, NSPC, CreAMINO^®^, and LYSOFORTE could be established in the diets of broiler chickens to improve the growth performance and economic efficiency.

## Background

There is a global demand for chicken meat which continues to increase exponentially [1]; this is partly due to the health claims associated with it, lack of cultural restrictions on its consumption, efficient production, and human population growth [2]. How this expected increase in poultry production will be achieved and what will be the consequences of this change for the sustainability of the production system are the main questions that need answer. The poultry production assured that more improvements in growth rate and store use efficacy can be achieved through genetic selection in the predictable future [3, 4]. As a consequence of the increased growth rate, birds reach slaughter weight at a later time than before. This has decreased the use of bird resources, and less energy is now needed to maintain body functions due to the shorter growth cycle [5–7]. This improvement in energy efficiency has resulted in a significant reduction in feed consumption of birds, thus improving the environmental sustainability of broiler production. However, it was reported that a higher energy level was more effective in body weight gain (BWG) and morphological parameters rather than the Cobb 500 broiler chicken recommendation, but in the case of feed conversion ratio, a more appropriate energy level was achieved from Cobb 500 broiler chicken recommendation [8].

New feeding practices for poultry include greater accuracy in providing nutrients for optimal growth performance and reducing feed costs and excess nutrients associated with environmental impact [9–14]. According to NRC [15], the standard measure for describing the requirements of energy for poultry and the energy content of diets is the metabolizable energy (ME). The ME is a costly part of poultry diets and it is considered a significant portion of the total cost of producing broiler chicken [16–18]. This supplemental energy requires ME optimization in the diets to decrease feed costs [19]. The level of dietary ME level has a significant role in adjusting the feed efficiency and feed intake in broilers [20]. Several trials have been done to increase the availability of energy for broiler and to raise the accuracy of determining the values of ME of dietary constituent [12, 21–26]. The study of Tasirnafas et al. [27] indicated that the highest weight was reported in the ostrich chicks fed on 10% dietary wastage and 2500 kcal/kg dietary energy.

Many feed additives like enzymes, emulsifiers, and creatine (CreAMINO^®^) have energy matrix value in poultry feed formulation. Nonstarch polysaccharides (NSPs) are indigestible carbohydrates that result in an increase in the viscosity of the gut digesta and a decrease in the nutrients’ availability for digestion due to nutrient enclosure. Nonstarch polysaccharides affect fat digestion more than other nutrients, and the saturated fatty acid digestion is affected more than the unsaturated fatty acids. To overcome the negative effects of these compounds on the growth performance, NSP-degrading enzymes are used. For instance, xylanases and β-glucanases are used for the degradation of the arabinoxylans and β-glucans in wheat, rye, barley, and oats and have demonstrated efficiency in improving the nutritional value of these grains for poultry [28]. Therefore, the most important role of supplying enzymes in monogastric animal diets is increasing the concentration of the apparent ME of the feed that consequently has economic benefits. This increase is usually in the range of 50–150 kcal/kg feed depending on the nature and quality of the ingredients used in the diet and the nature of the used enzyme [29]. NSP-degrading enzymes can reduce the increase in the viscosity of digesta resulting from sugars leaking from the grain cell walls by releasing the nutrients trapped in the feed cellular matrix [30] because the viscosity is larger in smaller birds and decreases with age.

Using an additional energy source in broiler diets represented in fats and oil supplementation is a common practice in the poultry industry. On the other hand, young chicks lack the necessary enzymes needed for effective digestion that improves with age with a significant improvement in the metabolic energy values of lipids in poultry from 1.5 to 3.5 weeks of age [31, 32]. The most convenient dietary tool to use is lysophospholipids, an absorption enhancer, which can increase emulsification and absorption of fats in the intestine and permit the remodeling of diets to be at the least cost without harming poultry performance [31]. Emulsifiers could play a key role in micelle formation, and lysophospholipids are natural surfactants of hydrolyzed soy lecithin [33].

Guanidinoacetic acid (GAA) is a readily available precursor of creatine, which is synthesized in the kidneys from glycine and L-arginine by L-arginine: glycine amidinotransferase. Next, GAA forms creatine in the liver by the action of guanidinoacetate *N*-methyltransferase. Creatine is a natural component that occurs in the tissues of the animal's body and acts as an important function in energy metabolism as an energy carrier in the cells [34]. It is estimated that about 50% of creatine required daily is synthesized by the animal whereas the rest must be supplemented by the diet daily. Short-term high-intensity exercise, or repeated bursts of exclusive power, can benefit from creatine supplementation; therefore, the sparing effect of creatine on energy and protein requirement is studied and it is concluded that it has a matrix value of 50 kcal/kg feed [35]. Furthermore, GAA may be essential for feeding poultry as a substitute for dietary arginine and to maintain the bird's total energy balance [36].

Therefore, this study aimed to investigate whether the energy matrix values of the NSP-degrading enzymes, emulsifier (LYSOFORTE^®^), and GAA (CreAMINO^®^) or their combinations could be summed or not by assessing the effects of their dietary supplementation on the broilers’ growth performance, carcass traits, and the economic value of the diets.

## Results

### Growth performance

The effects of NSP-degrading enzymes, LYSO, CreAMINO^®^, and their combinations with the reduced matrix values on the growth performance parameters are shown in Table 1. Throughout the starter stage, a significant increase (*P* < 0.05) in the growth performance parameters except for the FCR was reported in the birds fed on a diet supplemented with NSPL with its reduced energy matrix value in comparison with the control group. No significant effects (*P* > 0.05) were observed in the BW, BWG, FI, and FCR in the NSP-degrading enzymes, LYSO, CreAMINO^®^, NSPC, and NSPLC groups with their reduced energy matrix value in comparison with the control group. Throughout the grower stage, the BW and BWG were significantly higher (*P* < 0.05) only in the NSPL group with its reduced energy matrix value. The FI was not significantly affected (*P* = 0.39) by the treatments. The FCR was significantly lower (*P* < 0.05) in the NSPL and NSPC groups. During the finisher period, the dietary supplementation of the LYSO, CreAMINO^®^, NSPL, and NSPC with the reduced energy matrix values resulted in a significant increase (*P* < 0.05) in the bird's BW and BWG and a significant decrease (*P* < 0.05) in the FCR. The final BW, total BWG, and relative growth rate (RGR) were increased significantly (*P* < *0*.01) in this order NSPL >NSPC >CreAMINO^®^ > LYSO with the reduced energy matrix value. The overall FCR was decreased significantly (*P* = 0.00) in this order NSPC < CreAMINO^®^ < NSPL < LYSO with the reduced energy matrix value. No significant differences (*P* > 0.05) in the final BW, total BWG, RGR, and FCR in the NSP-degrading enzymes and NSPLC groups with their reduced energy matrix values in comparison with the control group. The total FI was not significantly differed (*P* > 0.05) by the treatments.

**Table 1:** The effects of dietary supplementation of NSP-degrading enzymes, LYSOFORTE^®^, CreAMINO^®^ and their combination on the growth performance of broiler chickens:

Parameter	T1	T2	T3	T4	T5	T6	T7	SEM	*P-value*
Int. Wt. (g)	42.34	42.98	42.50	43.24	43.28	43.20	43.44	0.13	0.36
Starter period									
BW (g)	423.65^bc^	437.60^ab^	415.09^c^	427.31^abc^	446.33^a^	429.99^abc^	421.60^bc^	2.88	0.05
BWG (g)	381.31^bc^	394.61^ab^	372.58^c^	384.07^abc^	403.04^a^	386.79^abc^	378.16^bc^	2.85	0.05
FI (g)	465.64^bc^	488.01^ab^	459.79^c^	464.02^bc^	496.15^a^	470.47^bc^	476.53^abc^	3.60	0.02
FCR	1.22	1.23	1.23	1.21	1.23	1.21	1.26	0.005	0.07
Grower period									
BW(g)	1073.15^bc^	1112.31^ab^	1034.32^c^	1109.44^abc^	1159.91^a^	1089.62^abc^	1065.89^bc^	10.02	0.02
BWG (g)	649.50^bc^	674.71^abc^	619.23^c^	682.12^ab^	713.58^a^	659.62^abc^	644.29^bc^	7.51	0.03
FI (g)	1062.82	1097.29	1037.18	1067.67	1118.65	1061.87	1073.78	9.14	0.39
FCR	1.63^ab^	1.62^ab^	1.67^a^	1.56^c^	1.57^c^	1.60^bc^	1.66^a^	0.008	0.001
Finisher period									
BW(g)	2188.32^d^	2275.40^ cd^	2363.88^bc^	2459.90^ab^	2519.21^a^	2491.28^ab^	2275.00^ cd^	22.72	0.001
BWG(g)	1115.17 ^b^	1163.08 ^b^	1329.56 ^a^	1350.46^a^	1359.30^a^	1401.66^a^	2258.22^b^	20.43	0.001
FI(g)	2256.98	2302.51	2150.40	2305.30	2365.53	2263.14	2217.22	24.97	0.34
FCR	2.03^a^	1.98^a^	1.63^bc^	1.70^bc^	1.74^bc^	1.61^c^	1.83^ab^	0.037	0.002
Overall performance									
BW(g)	2188.32^d^	2275.40^ cd^	2363.88^bc^	2459.90^ab^	2519.21^a^	2491.28^ab^	2275.00^ cd^	22.72	0.001
BWG(g)	2145.98^d^	2232.41^ cd^	2321.38^bc^	2416.66^ab^	2475.93^a^	2448.08^ab^	2231.56^ cd^	22.70	0.001
FI(g)	3785.45	3853.36	3707.48	3806.50	3980.34	3795.49	3767.54	34.65	0.47
FCR	1.76^a^	1.72^a^	1.60^bc^	1.57^c^	1.60^bc^	1.55 ^c^	1.68^ab^	0.01	0.001
RGR	192.40^c^	192.58^bc^	192.92^ab^	193.08^a^	193.24^a^	193.18^a^	192.50^bc^	0.06	0.001

^a,b,c,d^ Means within the same row carrying different superscripts are significantly different at (*P* < 0.05).

T1: Control group "basal diet with no additives (breeder recommendation (BR)). T2: Basal diet minus 100 kcal/kg supplemented with 0.02% NSP-degrading enzymes (NSP). T3: Basal diet minus 50 kcal/kg supplemented with 0.025% emulsifier (LYSOFORTE^®^). T4: Basal diet minus 50 kcal/kg supplemented with 0.06% guanidinoacetic acid (CreAMINO^®^). T5: Basal diet minus 150 kcal/kg supplemented with a mixture of NSP and LYSOFORTE^®^ (NSPL), T6: Basal diet minus 100 kcal/kg supplemented with a mixture of NSP and CreAMINO^®^ (NSPC). T7: Basal diet minus 200 kcal/kg supplemented with a mixture of NSP, LYSO, and CreAMINO^®^ (NSPLC)..

*BW* body weight; *BWG* body weight gain; *FI* feed intake; *FCR* feed conversion ratio; *RGR* relative growth rate

### Carcass traits

The effects of NSP-degrading enzymes, LYSOFORTE^®^, CreAMINO^®^, and their combinations with the reduced matrix values on the carcass traits relative to the live body weight (%) are summarized in Table 2. The results revealed that there were no significant differences (*P* > 0.05) in the weights of breast meat, carcass weight, drumstick, abdominal fat, thigh, liver, gizzard, and proventriculus as a result of the treatments. However, there was a numerical decrease in the abdominal fat % in LYSOFORTE^®^ and CreAMINO^®^ groups.

**Table 2:** The effect of dietary supplementation of NSP-degrading enzymes, LYSOFORTE^®^, CreAMINO^®^ and their combination on the weight of carcass traits relative to the live body weight (%):

Parameter	T1		T2	T3	T4	T5	T6	T7	SEM	*P-value*
Carcass weight		91.69	90.73	94.38	89.97	86.99	93.25	88.49	0.87	0.27
Breast meat		28.29	24.47	26.08	25.39	28.80	27.76	27.19	0.42	0.22
Thighs		9.70	9.66	9.41	10.17	9.24	9.52	9.84	0.19	0.97
Drumstick		9.71	10.64	9.89	11.84	10.83	10.35	13.79	0.41	0.18
Liver		2.29	1.95	2.09	2.60	1.79	2.64	2.34	0.11	0.52
Gizzard		1.59	1.54	1.72	1.79	0.89	1.83	1.56	0.12	0.67
Proventriculus		0.36	0.26	0.33	0.36	0.32	0.33	0.28	0.01	0.23
Abdominal Fat		1.82	1.60	1.39	1.16	1.62	2.04	1.47	0.09	0.39

^a,b,c^ Means within the same row carrying different superscripts are significantly different at (*P* < 0.05).

T1: Control group "basal diet with no additives (breeder recommendation (BR)). T2: Basal diet minus 100 kcal/kg supplemented with 0.02% NSP-degrading enzymes (NSP). T3: Basal diet minus 50 kcal/kg supplemented with 0.025% emulsifier (LYSOFORTE^®^). T4: Basal diet minus 50 kcal/kg supplemented with 0.06% guanidinoacetic acid (CreAMINO^®^). T5: Basal diet minus 150 kcal/kg supplemented with a mixture of NSP and LYSOFORTE^®^ (NSPL), T6: Basal diet minus 100 kcal/kg supplemented with a mixture of NSP and CreAMINO^®^ (NSPC). T7: Basal diet minus 200 kcal/kg supplemented with a mixture of NSP, LYSO, and CreAMINO^®^ (NSPLC).

### Economic importance

The economic importance of the diets is shown in Table 3. No significant differences (*P* > 0.05) were reported in feed costs and total costs between the control group and other groups. The total returns were significantly higher in birds fed on diets supplemented with NSPL, NSPC, CreAMINO^®^, and LYSO with the reduced energy matrix value (*P* < 0.05). The net profit and performance index were significantly increased (*P* < 0.01) in birds fed on diets supplemented with NSPC, NSPL, CreAMINO^®^, and LYSO with the reduced energy matrix value. The economic efficiency was increased significantly (*P* < 0.01) in birds fed on diets supplemented with CreAMINO^®^, NSPC, NSPL, and LYSO with the reduced energy matrix value. The cost of feed per kg of BWG was decreased (*P* < 0.01) in birds fed on diets supplemented with NSPC**,** NSPL, and CreAMINO^®^ with the reduced energy matrix value. The dietary supplementation with NSP-degrading enzymes and NSPLC with the reduced energy matrix value had no significant effect (*P* > 0.05) on the economic value of the diets.

**Table 3:** The effect of dietary supplementation of NSP-degrading enzymes, LYSO, CreAMINO^®^ and their combinations on the economic efficiency of the experimental diets:

Parameter	T1	T2	T3	T4	T5	T6	T7	SEM	*P-value*
Final BW (kg)	2.18^d^	2.27^ cd^	2.36^bc^	2.45^ab^	2.51^a^	2.49^ab^	2.27^ cd^	0.02	0.001
Feed cost (LE)	14.76	14.52	15.06	14.22	15.20	14.57	14.44	0.12	0.290
Total cost (LE)	22.26	22.02	22.56	21.72	22.70	22.07	21.94	0.12	0.290
Total return/bird(LE)	39.38^d^	40.95^ cd^	42.55^bc^	44.27^ab^	45.34^a^	44.84^ab^	40.95^ cd^	0.40	0.001
Net profit (LE)	17.12^c^	18.92^bc^	19.29^b^	22.55^a^	22.64^a^	22.76^a^	19.00^bc^	0.40	0.001
E. EF.^*^	1.16^c^	1.30^bc^	1.32^b^	1.58^a^	1.49^a^	1.56^a^	1.31^bc^	0.03	0.001
Feed cost/kg gain	6.89^a^	6.65^a^	6.74^a^	6.14^bc^	6.13^bc^	5.95^c^	6.47^ab^	0.07	0.001
PI (%) ^$^	124.39^c^	131.28^bc^	137.20^b^	153.79^a^	156.96^a^	160.54^a^	134.74^bc^	2.42	0.001

*Economic efficiency, ^$^ Performance index. LE: Egyptian pound.

^a,b,c,d^ Means within the same row carrying different superscripts are significantly different at (*P* < 0.05).

T1: Control group "basal diet with no additives (breeder recommendation (BR)). T2: Basal diet minus 100 kcal/kg supplemented with 0.02% NSP-degrading enzymes (NSP). T3: Basal diet minus 50 kcal/kg supplemented with 0.025% emulsifier (LYSOFORTE^®^). T4: Basal diet minus 50 kcal/kg supplemented with 0.06% guanidinoacetic acid (CreAMINO^®^). T5: Basal diet minus 150 kcal/kg supplemented with a mixture of NSP and LYSOFORTE^®^ (NSPL), T6: Basal diet minus 100 kcal/kg supplemented with a mixture of NSP and CreAMINO^®^ (NSPC). T7: Basal diet minus 200 kcal/kg supplemented with a mixture of NSP, LYSO, and CreAMINO^®^ (NSPLC).

## Discussion

Rapid growing poultry requires nitrogenous and energetic compounds to support performance and growth. However, contentious problems exist when feeding small birds with large amounts of nutrients [37, 38]. The current study had been done to assess the impact of dietary supplementation of 0.02% NSP-degrading enzymes, 0.025% emulsifier (LYSOFORTE^®^), and 0.06% creatine (CreAMINO^®^) and their combinations with the reduced energy matrix value on the growth performance of broilers, carcass traits, and the economic value of the diets. The results revealed that the NSPL, NSPC, CreAMINO^®^, and LYSO supplementation with the reduced energy matrix values (150, 100, 50, and 50 kcal/kg which are lower than the control group, respectively) enhanced the growth performance of the broilers, while matrix values of NSP-degrading enzymes alone or NSPLC did not result in a significant enhancement in the growth performance all over the experimental period. However, the dietary addition of NSP-degrading enzymes with LYSO or CreAMINO^®^ led to significantly better performance than that of the control group or the single supplementation. The final BW, BWG, and RGR were increased significantly in NSPL > NSPC > CreAMINO > LYSO with the reduced energy matrix value. The overall FCR was decreased in NSPC < CreAMINO < NSPL < LYSO with the reduced energy matrix value. Similarly, Kocher, et al. [39] reported a significant increase in AME and protein content of SBM-based diets by supplementing an enzyme compound containing multicarbohydrase activities without significant improvement of the birds’ growth performance. The results of Meng and Slominski [40] showed that supplementing the corn-SBM diet with a multicarbohydrase mixture of cell wall-degrading activities had no consequence on feed intake and BW but improved BWG and FCR. Zanella et al. [41] reported a significant improvement in the broilers’ growth performance by the supplementation of a marketable enzyme product containing amylase, protease, and xylanase to a corn-SBM diet due to the increase in the ileal digestibility of AME and protein.

The present study showed that the dietary supplementation with CreAMINO^®^ alone or in combination with NSP-degrading enzymes with the reduced energy matrix values improved the growth performance of broiler chickens all over the experimental periods. These results can be attributed to the GAA as a precursor to creatine synthesis because more arginine became obtainable for other functions as protein synthesis [42]. Guanidinoacetic acid supplementation has been shown to have sparing effects on arginine, increasing the availability of arginine in broilers [43]. In addition, arginine has an effect on the intestinal morphological features [44]. Therefore, the dietary inclusion of GAA may affect intestinal digestion and nutrient absorption. Fosoul et al. [26] reported improved performance of chickens fed low ME diet with supplemental GAA probably via improved dietary NEp. They suggested that including GAA in diets with lower energy content might improve the overall body energy retention such as fats or proteins which contribute to the well understanding of energy use in broiler chickens. Michiels et al. [45] reported an improved FCR and average daily gain all over the experimental period and increased breast meat yield in groups supplemented with GAA in comparison with the control birds. Also, Lemme et al. [46] reported that guanidinoacetic acid (GAA) resulted in improvement of the bird performance and it was optimal between 0.06% and 0.12% supplemented GAA due to the vital role of GAA supplementation in energy metabolism that increased the muscle concentration of creatine and creatine phosphate: ATP ratio. Abudabos et al. [35] reported a significant improvement in FCR all over the experimental periods by supplementing the low-energy diets with CreAMINO^®^, and the greatest performance was recorded for the diet with 50 kcal/kg lower ME. Dilger et al. [36] reported similar results of the gain and gain/feed when GAA and creatine were added to the semipurified and practical-type diets.

Various factors influence the lipid digestibility in birds, and these include the saturation degree [47], the free fatty acids levels [48], and the position of the fatty acid in the triglyceride molecule [49]. Also, fat digestion may be influenced by metabolic and physiological factors that depend on the bile salts’ surfactant action for the emulsification process. Though baby chicks have inadequate amounts of bile salts resulting in poor fat utilization, this significantly enhanced fat utilization from 1.5 to 3.5 weeks of age [32, 50–52]. Therefore, many experiments have interested in supplementing emulsifiers or bile salts to enhance fat utilization. Our results revealed a nonsignificant effect of LYSOFORTE^®^ on the growth performance of broilers during the starter stage but it had an improving effect on the overall performance. However, the combination of NSP-degrading enzymes and LYSOFORTE^®^ improved the BW and BWG throughout the starter stage and all over the period. These positive effects may be due to the increase in the digestibility of fatty acids [53]. LYSOFORTE^®^ formed smaller and more stable micelles than other emulsifiers such as lecithin [54], and this is a major factor influencing the absorption of lipid and lipophilic substances [55]. Lysophospholipids have been reported to improve intestinal permeability to macromolecules such as dextrans and proteins [56], affect the protein channels formation [57], regulate the activity of various enzymes [58], and cause hypertrophy of the epithelial cells in broiler duodenum [59]. Zhang et al. [53] showed that LYSOFORTE^®^ supplementation resulted in an improvement in growth performance during the starter period. Melegy et al. [31] reported an improving effect of LYSOFORTE^®^ Booster to the lowered performance induced by a negative control diet. The study of Jansen et al. [60] showed a high ME due to the utilization of lysophospholipids in the feed of broiler chickens. Zhao and Kim [61] showed increased BWG and lower FCR in broiler chickens fed diets supplemented with lysophospholipids compared to nonsupplemented diets. Also, a low-energy diet supplemented with emulsifier and multienzymes partially improved the growth performance and hence correct the depressing effects of the low-energy diets in broilers [62, 63]. Zampiga et al. [64] suggested that the use of lysophospholipids can improve the feed efficiency of broiler chickens.

The improvements of NSPL, NSPC, LYSO, and CreAMINO^®^ supplementation on the performance of broilers were reflected in the economic value of the diets where the total return was increased in NSPL, NSPC, CreAMINO^®^, and LYSO. The net profit and performance index were significantly increased in NSPC, NSPL, CreAMINO^®^, and LYSO. The economic efficiency was increased in CreAMINO^®^, NSPC, NSPL, and LYSO with the reduced energy matrix value. The cost of feed per kg of BWG was decreased significantly in NSPC, NSPL, and CreAMINO^®^ with the reduced energy matrix value. Similar results were obtained in the study of Amer, et al. [12] who reported improved economic efficiency by supplementing the low-energy diet with the alpha-galactosidase enzyme.

Regarding the effect of dietary supplementation of NSP-degrading enzymes, LYSOFORTE^®^, CreAMINO^®^, and their combinations with the reduced matrix values on the carcass traits, the results revealed nonsignificant effects of these additives on the relative weights of breast meat, carcass weight, drumstick, thigh, liver, gizzard, and proventriculus. There was a numerical decrease in the abdominal fat weight in LYSOFORTE^®^ and CreAMINO^®^ groups. Similarly, Zhao and Kim [61] reported decreased abdominal fat weight in lysophospholipid-supplemented groups compared to the basal diet. Also, Zhang [65] reported decreased abdominal fat percentage in broiler chickens fed diets that contained varying levels of emulsifiers at 0.02, 0.035, 0.05, and 0.065% on day 42 which give evidence that emulsifiers can increase the body fat cycle and improve the deposition of muscle fats. Zampiga et al. [64] showed that lysophospholipids had a limited effect on carcass traits of broiler chickens. The results of Metwally et al. [66] showed increased carcass dressing and decreased abdominal fat as a result of CreAMINO^®^ supplementation in broiler chicken diets.

## Conclusions

Dietary supplementation with CreAMINO^®^ or LYSOFORTE^®^ or the mixture of NSP-degrading enzyme with CreAMINO^®^ or with LYSOFORTE^®^ with the reduced energy matrix value of 50, 50, 100, and 150 kcal/kg lower than the breeder recommendation, respectively, can improve the final body weight, body weight gain, feed conversion ratio, and the relative growth rate of broiler chickens and can increase the economic efficiency of their supplemented diets.

## Methods

### Birds

A total of 525-one-day-old broiler chicks (Ross 308 broiler) with an initial body weight of 42.96 ± 0.87 g were obtained from a commercial chick producer (Dakahlia Poultry, Mansoura, Egypt) and used in the experiments. The experiment was carried out at the experimental station of the Faculty of Veterinary Medicine, Zagazig University, Egypt. The experimental protocol was approved by the Ethics of the Institutional Animal Care and Use Committee of Zagazig University, Egypt (ZUIACUC–2019), and all animal experiments were performed in accordance with the recommendations described in “The Guide for the Care and Use of Laboratory Animals in Scientific Investigations”. The replicate number and treatment group were visible at each pen. The maximum number of birds per pen and space per bird, respectively, followed the current legislation. The birds were housed in pens with 15 birds each. Feed and water were supplied for ad libitum consumption. Pens were equipped with feeders and drinkers. The temperature and lighting regime followed the breeder's recommendation. All pens were checked daily for general health status and whether there were sick and dead birds. Standard health and vaccination practices were done against New Castle (at the 4th and 14th days) and Gumboro diseases (at the 7th and 22 days). After the study, all remaining birds were released.

### Investigated feed additives

The used feed additives were as follows: (1) nonstarch polysaccharide- (NSP-) degrading enzymes, in a commercial form AveMix^®^ XG 10 (AVEVE Biochem, Merksem, Belgium), which is a multienzyme concept with high glucanase and high xylanase activity, designed for monogastric feeds (active substances: endo–1,3 (4)-ß-glucanase; endo-1,4-ß-xylanase and cellulase); (2) bioemulsifier in a commercial form LYSOFORTE^®^ (Kemin Industries, Inc., USA); and (3) GAA in a commercial form CreAMINO^®^ (AlzChem, Trostberg, Germany).

### Diets and experimental design

Birds were randomly allocated into seven treatments with five replicates (15 chicks/ replicate). The seven treatments are as follows: (1) basal diet with no additives (breeder recommendation) which is the control group, (2) basal diet minus 100 kcal/kg supplemented with 0.02% NSP-degrading enzymes (NSP), (3) basal diet minus 50 kcal/kg supplemented with 0.025% emulsifier (LYSOFORTE^®^), (4) basal diet minus 50 kcal/kg supplemented with 0.06% guanidinoacetic acid (CreAMINO^®^), (5) basal diet minus 150 kcal/kg supplemented with a mixture of NSP-degrading enzymes and LYSOFORTE^®^ (NSPL), (6) basal diet minus 100 kcal/kg supplemented with a mixture of NSP-degrading enzymes and CreAMINO^®^ (NSPC), and (7) basal diet minus 200 kcal/kg supplemented with a mixture of NSP-degrading enzymes, LYSO, CreAMINO^®^, and NSPLC. The experiment lasted for 35 d. The experiment was divided into three periods including the starter period (0–10 d), the grower period (11–22 d), and the finisher period (23–35 d). The proximate composition and chemical composition of the experimental diets are shown in (Tables 4 and 5) based on a corn-soybean meal, and they were formulated to follow the Ross Manual Guide [67]. The feed was offered to the birds in pelleted physical feed form in the whole period (starter, grower, and finisher).

**Table 4:** The proximate composition of the experimental diets (%)

Ingredients	Starter period (0-10d)							Grower period (11-22d)							Finisher period (23-35d)						
	T1	T2	T3	T4	T5	T6	T7	T1	T2	T3	T4	T5	T6	T7	T1	T2	T3	T4	T5	T6	T7
Corn	55.8	56.5	56.8	57.03	55.97	55.94	55.39	64.34	64.88	64.84	63.78	63.75	60.04	67.70	68.80	68.72	69.28	66.68	66.77	67.21	64.34
Soybean meal, 48%CP	28.7	32	28.7	27.99	35.92	35.80	39.69	23.77	27.95	24.02	23.90	31.82	31.72	35.43	21.33	23.65	21.27	19.67	27.57	27.43	28.99
Corn gluten meal, 60% CP	9.9	6.9	9.8	10.27	3.65	3.76	0.52	6.85	3.22	6.50	6.61	0	0.09	0	5.38	3.24	5.28	6.57	1.50	1.50	0
Soybean oil	1.63	1.64	1.63	1.63	1.65	1.64	1.65	1.80	1.00	1.00	1.00	1.00	1.00	1.00	2.22	1.00	1.34	1.01	1.00	1.00	0.84
Dicalcium phosphate	1.11	1.08	1.11	1.12	1.05	1.05	1.02	1.39	1.40	1.39	1.39	1.41	1.41	1.39	1.25	1.25	1.24	1.24	1.24	1.24	1.25
Limestone	0.005	0.005	0.005	0.01	0.01	0.01	0.01	0.91	0.88	0.91	0.91	0.85	0.85	0.82	0.90	0.88	0.90	0.91	0.85	0.85	0.50
L-Lys-HCl	1.5	0.5	0.6	0.50	0.50	0.50	0.50	0.32	0.22	0.32	0.32	0.12	0.12	0.01	0.30	0.30	0.30	0.30	0.30	0.30	0.30
Premix ^a^	0.3	0.3	0.3	0.30	0.30	0.30	0.30	0.30	0.30	0.30	0.30	0.30	0.30	0.30	0.28	0.22	0.28	0.32	0.11	0.12	0.08
Sodium bicarbonate	0.18	0.21	0.18	0.17	0.24	0.24	0.27	0.23	0.18	0.23	0.23	0.15	0.14	0.51	0.21	0.23	0.22	0.20	0.27	0.27	0.28
Salt (NaCl)	0.25	0.21	0.25	0.26	0.17	0.17	0.13	0.20	0.24	0.20	0.20	0.27	0.27	0.19	0.21	0.19	0.21	0.23	0.25	0.25	0.25
DL-Methionine	0.4	0.31	0.4	0.41	0.21	0.21	0.12	0.18	0.21	0.18	0.18	0.23	0.23	0.20	0.17	0.18	0.17	0.16	0.18	0.18	0.19
Therionine	0.21	0.23	0.21	0.21	0.25	0.25	0.28	0.05	0.04	0.05	0.05	0.03	0.03	0	0.04	0.04	0.04	0.04	0.01	0.01	0.01
Phytase Enzyme	0.05	0.04	0.05	0.05	0.04	0.04	0.03	0.01	0.01	0.01	0.01	0.01	0.01	0.01	0.01	0.01	0.01	0.01	0.01	0.01	0.01
NSP-degrading enzymes	-	0.02	-	-	0.02	0.02	0.02	-	0.02	-	-	0.02	0.02	0.02	-	0.02	-	-	0.02	0.02	0.02
LYSOFORTE®	-	-	0.025	-	0.025	-	0.025	-	-	0.025	-	0.025	-	0.025	-	-	0.025	-	0.025	-	0.025
CreAMINO®	-	-	-	0.06	-	0.06	0.06	-	-	-	0.06	-	0.06	0.06	-	-	-	0.06	-	0.06	0.06

^a^ Premix per kg of diet: vitamin A, 1 500 IU; vitamin D3, 200 IU; vitamin E, 10 mg; vitamin K3, 0.5 mg; thiamine, 1.8 mg; riboflavin, 3.6 mg; pantothenic acid, 10 mg; folic acid, 0.55 mg; pyridoxine, 3.5 mg; niacin, 35 mg; cobalamin, 0.01 mg; biotin, 0.15 mg; Fe, 80 mg; Cu, 8 mg; Mn, 60 mg; Zn, 40 mg; I, 0.35 mg; Se, 0.15 mg.

T1: Control group "basal diet with no additives (breeder recommendation (BR)). T2: Basal diet minus 100 kcal/kg supplemented with 0.02% NSP-degrading enzyme (NSP). T3: Basal diet minus 50 kcal/kg supplemented with 0.025% emulsifier (LYSOFORTE^®^). T4: Basal diet minus 50 kcal/kg supplemented with 0.06% guanidinoacetic acid (CreAMINO^®^). T5: Basal diet minus 150 kcal/kg supplemented with a mixture of NSP and LYSOFORTE^®^ (NSPL), T6: Basal diet minus 100 kcal/kg supplemented with a mixture of NSP and CreAMINO^®^ (NSPC). T7: Basal diet minus 200 kcal/kg supplemented with a mixture of NSP, LYSO, and CreAMINO^®^ (NSPLC).

**Table 5:** The proximate chemical composition of the experimental diets (%)

	Starter period (0-10d)							Grower period (11-22d)							Finisher period (23-35d)						
	T1	T2	T3	T4	T5	T6	T7	T1	T2	T3	T4	T5	T6	T7	T1	T2	T3	T4	T5	T6	T7
Dry Matter	88.63	88.50	88.51	88.52	88.47	88.50	88.47	88.61	88.50	88.49	88.52	88.47	88.50	88.49	88.64	88.48	88.51	88.50	88.46	88.48	88.40
Crude Protein	24.70	24.48	24.69	24.86	24.24	24.38	24.14	21.09	20.82	21.06	21.20	20.58	20.72	22.00	19.27	19.12	19.27	19.49	19.64	19.72	19.56
Crude Fiber	3.07	3.26	3.09	3.06	3.45	3.44	3.63	2.93	3.15	2.96	2.96	3.34	3.34	3.48	2.85	3.00	2.87	2.80	3.17	3.16	3.25
Fat	4.68	3.62	3.83	3.74	3.49	3.50	3.37	5.04	4.13	4.26	4.26	4.01	4.01	3.94	5.47	4.22	4.63	4.36	4.12	4.12	3.58
Ash	6.12	6.27	6.13	6.11	6.42	6.42	6.57	5.43	5.60	5.44	5.44	5.76	5.75	6.19	5.13	5.23	5.13	5.07	5.49	5.49	5.57
AME (kcal/kg)	3024.9	3025	3024.9	3024.9	3025	3024.9	3025	3100	3100	3100	3100	3100	3099.9	3100	3150	3150	3150	3149.9	3149.9	3150	3150
Calcium	1.05	1.05	1.05	1.05	1.05	1.05	1.05	0.90	0.90	0.90	0.90	0.90	0.90	0.90	0.85	0.85	0.85	0.85	0.85	0.85	0.85
Phosphrus	0.77	0.78	0.77	0.77	0.79	0.79	0.80	0.70	0.71	0.71	0.71	0.72	0.72	0.73	0.66	0.67	0.67	0.66	0.68	0.68	0.69
Available Phosphrus	0.50	0.50	0.50	0.50	0.50	0.50	0.50	0.45	0.45	0.45	0.45	0.45	0.45	0.45	0.42	0.42	0.42	0.42	0.42	0.42	0.42
Lysine	1.38	1.38	1.38	1.38	1.38	1.38	1.39	1.17	1.17	1.17	1.17	1.18	1.18	1.18	1.06	1.06	1.06	1.06	1.07	1.07	1.07
Methionine	0.62	0.62	0.62	0.62	0.62	0.62	0.63	0.53	0.54	0.53	0.53	0.54	0.54	0.52	0.49	0.49	0.49	0.49	0.48	0.49	0.49
Cystine	0.40	0.39	0.40	0.40	0.39	0.39	0.38	0.35	0.34	0.35	0.35	0.33	0.33	0.35	0.33	0.32	0.33	0.33	0.33	0.33	0.32
Threonine	0.95	0.94	0.95	0.95	0.94	0.94	0.94	0.81	0.81	0.81	0.81	0.81	0.81	0.83	0.74	0.74	0.74	0.74	0.74	0.74	0.74

T1: Control group "basal diet with no additives (breeder recommendation (BR)). T2: Basal diet minus 100 kcal/kg supplemented with 0.02% NSP-degrading enzyme (NSP). T3: Basal diet minus 50 kcal/kg supplemented with 0.025% emulsifier (LYSOFORTE^®^). T4: Basal diet minus 50 kcal/kg supplemented with 0.06% guanidinoacetic acid (CreAMINO^®^). T5: Basal diet minus 150 kcal/kg supplemented with a mixture of NSP and LYSOFORTE^®^ (NSPL), T6: Basal diet minus 100 kcal/kg supplemented with a mixture of NSP and CreAMINO^®^ (NSPC). T7: Basal diet minus 200 kcal/kg supplemented with a mixture of NSP, LYSO, and CreAMINO^®^ (NSPLC).

### Growth performance

The initial body weight of the one-day-old chick was recorded on arrival, and then birds were weighed at the end of each stage at 11, 22, and 35th days of age. Bodyweight gain was calculated as the difference between the final body weight during the intended period and the initial weight during the same period. Feed intake of each replicate was recorded as the difference between the feed offered weight and residues left and then divided by the number of birds in each replicate to determine the average feed intake per bird. The feed conversion ratio (FCR) was estimated at the end of each stage [68]: FCR = amount of feed consumed (g)/BWG (g). The relative growth rate (RGR) was calculated at the end of the experiment using the following equation [69]:

$$\mathrm{RGR}= [(\mathrm{W}2-\mathrm{W}1)/((\mathrm{W}1+\mathrm{W}2))/2]\times 100$$

### Carcass traits

At the end of the experiment, five birds per group (one bird /replicate) were randomly selected and fasted for 12 h with a free water supply, and then euthanized by cervical dislocation according to the American Veterinary Medical Association guidelines for the euthanasia of animals, AVMA [70] for carcass evaluation (e.g., carcass weight, breast meat weight, and abdominal fat pad).

### Economic importance

Collective efficiency measures were calculated according to El-Telbany and Atallah [71] and Dunning and Daniels [72]. They include the total return, total costs, variable costs, and net profit. The performance index (PI) was calculated according to North and Bell [73].

Total return (LE)/bird = price of kg x live body weight/bird.

Total costs (LE) = fixed costs + variable costs (feed cost, LE).

Net profit (LE) = total returns – total costs.

Economic efficiency (EEF) = net profit (LE)/total feed cost (LE).

Feed cost/kg gain = total feed cost/total weight gain.

Performance index % (PI) = final live body weight (kg)/feed conversion × 100.

### Statistical analysis

Shapiro–Wilk’s test was used to verify the normality and Levene’s test was used to verify homogeneity of variance components between experimental treatments and the assumption were achieved (p > 0.05). One-Way ANOVA was used to investigate the effect of dietary supplementation of NSP-degrading enzymes (NSP), emulsifier (LYSOFORTE^®^), and creatine (CreAMINO^®^) and their combinations with the reduced energy matrix value on the broilers’ growth performance, carcass traits, and the economic value of the diets using SPSS version 17 for Windows (SPSS, Inc., Chicago, IL, USA). The sample size used in this study was determined in line with existing research [12]. Duncan’s multiple range test was used to compare the differences between the means test [74] and the variation in the data was expressed as pooled SEM, and the significance level was set at *P* < 0.05.

## Data Availability

The datasets used and analyzed during the current study are available from the corresponding author on reasonable request.

## Abbreviations

NSP: Nonstarch polysaccharides
LYSO: LYSOFORTE^®^
Creatine: CreAMINO^®^
NSPL: A mixture of NSP and LYSOFORTE^®^
NSPC: A mixture of NSP and CreAMINO^®^
NSPLC: A mixture of NSP, LYSO, and CreAMINO^®^
GAA: Guanidinoacetic acid
ME: Metabolizable energy
BW: Body weight
BWG: Body weight gain
FI: Feed intake
FCR: Feed conversion ratio
RGR: Relative growth rate
LE: Egyptian pound
