# Correction to: Are the energy matrix values of the different feed additives in broiler chicken diets could be summed?

**DOI:** 10.1186/s12917-020-02712-w

**Published:** 2020-12-21

**Authors:** Abdallah E. Metwally, Ahmed A. A. Abdel-Wareth, Ahmed A. Saleh, Shimaa A. Amer

**Affiliations:** 1 Department of Nutrition and Clinical Nutrition, Faculty of Veterinary Medicine, Zagazig University, Zagazig, 44511 Egypt; 2 Department of Animal and Poultry Production, Faculty of Agriculture, South Valley University, Qena, 83523 Egypt; 3 Department of Poultry Production, Faculty of Agriculture, Kafr Elsheikh University, Kafr Elsheikh, 33516 Egypt

**Correction to: BMC Vet Res (2020) 16:391**

**https://doi.org/10.1186/s12917-020-02600-3**

Following the publication of the original manuscript [1], errors were determined in the data of Table 4. The corrected table can be viewed ahead.

**Table 4:** The proximate composition of the experimental diets (%)

Ingredients	Starter period (0-10d)							Grower period (11-22d)							Finisher period (23-35d)						
	T1	T2	T3	T4	T5	T6	T7	T1	T2	T3	T4	T5	T6	T7	T1	T2	T3	T4	T5	T6	T7
Corn	55.8	56.5	56.8	57.03	55.97	55.94	55.39	63.99	64.34	64.88	64.84	63.78	63.75	60.14	67.70	68.80	68.72	69.28	66.68	66.77	67.21
Soybean meal, 48 %CP	28.7	32.0	28.7	27.99	35.92	35.80	39.69	23.77	27.95	24.02	23.90	31.82	31.72	35.43	21.33	23.65	21.27	19.67	27.57	27.43	28.99
Corn gluten meal, 60% CP	9.90	6.90	9.80	10.27	3.65	3.76	0.52	6.85	3.22	6.50	6.61	0.00	0.09	0.00	5.38	3.24	5.28	6.57	1.50	1.50	0.00
Dicalcium Phosphate	1.63	1.64	1.63	1.63	1.65	1.64	1.65	1.39	1.40	1.39	1.39	1.41	1.41	1.39	1.25	1.25	1.24	1.24	1.24	1.24	1.25
Limestone	1.11	1.08	1.11	1.12	1.05	1.05	1.02	0.91	0.88	0.91	0.91	0.85	0.85	0.82	0.90	0.88	0.90	0.91	0.85	0.85	0.84
Phytase Enzyme	0.005	0.005	0.005	0.005	0.005	0.005	0.005	0.005	0.005	0.005	0.005	0.005	0.005	0.005	0.005	0.005	0.005	0.005	0.005	0.005	0.005
Soybean oil	1.50	0.50	0.60	0.50	0.50	0.50	0.50	1.80	1.00	1.00	1.00	1.00	1.00	1.00	2.22	1.00	1.34	1.01	1.00	1.00	0.50
Premix *	0.30	0.30	0.30	0.30	0.30	0.30	0.30	0.30	0.30	0.30	0.30	0.30	0.30	0.30	0.30	0.30	0.30	0.30	0.30	0.30	0.30
Salt (NaCl)	0.18	0.21	0.18	0.17	0.24	0.24	0.27	0.20	0.24	0.20	0.20	0.27	0.27	0.29	0.21	0.23	0.22	0.20	0.27	0.27	0.28
Sodium bicarbonate	0.25	0.21	0.25	0.26	0.17	0.17	0.13	0.23	0.18	0.23	0.23	0.15	0.14	0.21	0.21	0.19	0.21	0.23	0.25	0.25	0.25
L-Lysine HCl	0.4	0.31	0.4	0.41	0.21	0.21	0.12	0.32	0.22	0.32	0.32	0.12	0.12	0.11	0.28	0.22	0.28	0.32	0.11	0.12	0.08
DL-Methionine	0.21	0.23	0.21	0.21	0.25	0.25	0.28	0.18	0.21	0.18	0.18	0.23	0.23	0.20	0.17	0.18	0.17	0.16	0.18	0.18	0.19
Therionine	0.05	0.04	0.05	0.05	0.04	0.04	0.03	0.05	0.04	0.05	0.05	0.03	0.03	0	0.04	0.04	0.04	0.04	0.01	0.01	0.01
NSP Enzyme	-	0.02	-	-	0.02	0.02	0.02	-	0.02	-	-	0.02	0.02	0.02	-	0.02	-	-	0.02	0.02	0.02
LYSOFORTE®	-	-	0.025	-	0.025	-	0.025	-	-	0.025	-	0.025	-	0.025	-	-	0.025	-	0.025	-	0.025
CreAMINO®	-	-	-	0.06	-	0.06	0.06	-	-	-	0.06	-	0.06	0.06	-	-	-	0.06	-	0.06	0.06

*Premix per kg of diet: vitamin A, 1 500 IU; vitamin D3, 200 IU; vitamin E, 10 mg; vitamin K3, 0.5 mg; thiamine, 1.8 mg; riboflavin, 3.6 mg; pantothenic acid, 10 mg; folic acid, 0.55 mg; pyridoxine, 3.5 mg; niacin, 35 mg; cobalamin, 0.01 mg; biotin, 0.15 mg; Fe, 80 mg; Cu, 8 mg; Mn, 60 mg; Zn, 40 mg; I, 0.35 mg; Se, 0.15 mg

T1: Control group "basal diet with no additives (breeder recommendation (BR)). T2: Basal diet minus 100 kcal/kg supplemented with 0.02% NSP-degrading enzyme (NSP). T3: Basal diet minus 50 kcal/kg supplemented with 0.025% emulsifier (LYSOFORTE®). T4: Basal diet minus 50 kcal/kg supplemented with 0.06% guanidinoacetic acid (CreAMINO®). T5: Basal diet minus 150 kcal/kg supplemented with a mixture of NSP and LYSOFORTE® (NSPL), T6: Basal diet minus 100 kcal/kg supplemented with a mixture of NSP and CreAMINO® (NSPC). T7: Basal diet minus 200 kcal/kg supplemented with a mixture of NSP, LYSO, and CreAMINO® (NSPLC)
